# Progress in Surgery

**Published:** 1896-11-07

**Authors:** 


### PROGRESS IN SURGERY.

### HEAD INJURIES.

There are few surgical maxims more frequently quoted than that to the effect that "no injury of the head is ever so slight as to be despised, or ever so severe as to be despaired of." It is as true to-day as when it was first expressed; indeed, the practice of aseptic surgery has only served to extend and emphasise the truth of the latter part of the saying. In every medical journal we take up we find recorded cases illustrative of both sides of the subject. Cowan (*Intercolon. Med. Journ. of Australia*, May, 1896) reports a case of a miner who, while at work, was stunned by something falling on him from above. Beyond an abrasion on the right side of the head, slight giddiness, and some numbness and stinging in the left leg, he felt very little the worse, and desired to resume his work. Having been dissuaded from doing so he went home, but not for some hours after did he begin to show signs of serious intra-cranial mischief—vomiting, restlessness, pain in the head, followed by unconsciousness and coma, supervening within eighteen hours of the accident. The clinical signs pointed to a hæmorrhage, most likely from the middle meningeal artery, about the centre of the motor area, as the probable lesion. On trephining this was confirmed, and a large clot was removed, but bleeding continued, and could only be arrested by plugging. Subsequently complete recovery took place, and the patient returned to his work without any permanent ill effects.

As a case showing the other side of the subject may be quoted that of Percy Smith (*N. Y. Med. Jour.*, June, 1896). A boy, aged ten, was kicked on the forehead by a horse, sustaining a compound fracture of the frontal bone, three inches long, above the right supra-orbital ridge. Brain tissue projected through the fracture, and brain *débris* was scattered over the boy's hat. The pulse was scarcely perceptible, and only returned in response to drugs. Consciousness was not quite lost, and there was no sign of paralysis. Chloroform was administered, and the bone sufficiently elevated to permit of purification of the wound and the insertion of a drainage tube. Complete recovery took place; the boy returned to school in a few months, and according to his teacher showed no sign of cerebral deficiency. These cases are not quoted here as being unusual, but simply as types of a fairly common experience, and as indicating the encouragement there is for prompt and thorough surgical interference.

**Cases of Head Injury Presenting Unusual Symptoms.**—No two head cases are ever exactly alike, and some very interesting variations as to symptoms and pathological lesions are met with, *e.g.*, (1) Cowan reports a case where a man was injured by the capsizing of a cart. Among other evidences of basal fracture were bleeding from the nose and right ear, the latter only coming on two hours after the injury. There was also observed a very extensive subconjunctival ecchymosis and slight but continuous bleeding from the eye. The loss of blood appeared to act beneficially, as the patient soon regained consciousness, and made a satisfactory recovery. Absolute deafness of the right ear persisted even after the membrana tympani had healed, and pointed to an internal ear lesion. The vision of the left eye was so deficient that six weeks after the injury he could only read great primer type (No. 12), but it gradually improved so far as to enable him to read minion type (No. 4).

2. A case illustrative of another unusual symptom is reported by Leclère (*Deutsch. Med. Wochensch.*, May, 1896). A patient was struck on the vertex of the skull, and sustained a compound fracture over the position of the superior longitudinal sinus. Temporary unconsciousness, severe headache, and formication of the legs were followed by complete paralysis of both legs, the arms and face remaining unaffected. Bilateral clots were diagnosed and removed by operation, and in six weeks the patient was able for light work. Localised lesions of similar cortical centres on both sides of the brain are rare.

3. In a case reported by Scudder (*Amer. Jour. Med. Sc.*, Aug., 1896), a boy of nine, after being struck by a falling brick and having his skull fractured and depressed, showed complete motor paralysis of the left arm and leg, ptosis of the right lid, and dilatation of the right pupil. There was no sensory disturbance, and the patient was perfectly conscious throughout. Elevation of the fragments by operation resulted in perfect recovery.

4. Brandenburger (*Arch. f. Augenheil Kunde. Bd.* xxxi.) reports a case where the movements of the eye downwards and inwards, as well as upwards and outwards, were interfered with by a fragment of the orbital margin being driven in and impacted in the roof of the orbital cavity. Its removal was followed by recovery of the movements of the eye.

5. In the case of a man aged fifty-nine, reported by Oliver (*Med. Record*, May, 1896), left ptosis, hæmorrhage from the left nostril, and motor aphasia were each found on *post-mortem* examination to be due to separate lesions: the first to a fracture of the left orbital plate, the second to an independent fracture of the ethmoid, and the last to a middle meningeal hæmorrhage.

6. In the following case, recorded by Shepherd (*Br. Med. Journ.*, April, 1896), the seat of the lesion was unusual, and further interest is lent to the case by the development of certain symptoms which were apparently the result of a necessary step in the treatment. A man of thirty was thrown from his bicycle while descending a very steep hill. Temporary unconsciousness followed, and there was extensive bruising of the external parts and a slightly depressed fracture of the anterior-superior part of the left parietal bone. After being dressed he was comfortable and conscious, and he passed a good night. In the morning he became restless, stupid, slightly aphasic, and developed some right-sided paresis. Middle meningeal hæmorrhage was diagnosed and treated by trephining. The artery was found torn close down to the base of the skull, and the removal of the clot gave rise to fresh bleeding, copious in amount, and only controllable by ligature of the common carotid artery. After this had been done, to check venous oozing, the wound was packed with gauze. Two days later the patient was much improved, but on changing the dressing bleeding began again, and necessitated fresh packing. This caused great pain in the head and over the left eye and later complete motor aphasia. As the gauze was gradually removed day by day the pressure symptoms disappeared, and the writer is inclined to attribute them to the presence of the dressing rather than to the ligature of the common carotid artery. The patient made a complete recovery.

**Traumatic Epilepsy.**—Although it is true that the number of cases of traumatic epilepsy subjected to operative treatment is not so great at present as it was a few years ago, there are still many surgeons who are willing to give the patients even the temporary benefit which so often follows the operation. Amongst recently reported cases are the following:—

1. That of a man, aged thirty-three, recorded by Heaton (*Birmingham Med. Rev.*, June, 1896), who, sixteen months after receiving a compound depressed fracture of the vertex of the skull, suffered from aphasia, loss of power in the right arm and face, and frequent epileptiform convulsions. These last were both severe and numerous. During eighteen days he had no fewer than 1,147 distinct fits, regaining complete consciousness between the attacks. That the lesion was in, or over, the lower half of the ascending frontal and parietal convolutions and the hinder extremity of the third frontal convolution of the left side was indicated by aphasia, with complete paralysis of the muscles of the face, tongue, and arm of the right side, and the fact that each fit commenced by the twitching of the muscles of the right eye and right side of the face. The old scar, however, was above and in front of the cortical area involved. As the seat of disease was not reached by a first operation, a second one was performed, and the bone was found much thickened and altered in structure, and pressing on the cortex in the areas above mentioned. All convulsive attacks stopped after the operation, and speech and motor power gradually returned. Muscular sense and co-ordination were regained more slowly.

2. Weissgesber (*Ep. B. M. J.*, May, 1896) reports two cases operated upon. In the first, a boy when eighteen months old was struck on the head, and eight and a half years later began to suffer from Jacksonian fits. After six months' illness a mass of cicatricial tissue, and the bone, in which there was a deficiency, were removed by operation, with the result that during three years the boy had only one fit, and that three years after operation.

The second case was that of a man, aged twenty-eight, who developed signs of fæcal epilepsy seven years after an injury to his head. The fits became very frequent, and, on operating, a depression was found in the bone, and a meningeal cyst lay under it. The improvement in this case was only partial and temporary.

3. Armstrong's (*Montreal Med. Jour.*, May, 1896) case is somewhat similar as to history and clinical signs. A deficiency was found in the bone in the region of an old depression, and beneath the gap in the skull two thin-walled cysts were found. These were incised and drained, and there was a cessation of the fits. Only a few months elapsed between the conclusion of treatment and the publication of the case, however.

4. Howard's (*Intercol. Med. Jour.*, Australia, May, 1896) case refers to a girl of seventeen who was injured about the head when six years old. Fits, varying in severity, type, and frequency, occurred from the time of the injury onwards. As these were supposed to be due to a cortical lesion (most probably sclerosis) of the left motor region an operation was peformed, but without definite morbid findings or therapeutic results.

5. Another case, in which return of fits followed an operation for Jacksonian epilepsy, is reported by Oliver (*Med. Rec.*, May, 1896).

**Insanity following Head Injury.**—A case is reported by Dunham (*Amer. Med.-Surg. Bulletin*, Jan., 1896) of a woman in whose history "there was a strain of lunacy and also of epilepsy," who fell down a stair injuring her head. She suffered from symptoms indicative of compression, and gradually improved after the skull was opened. Subsequently she became quite insane, and had to be removed to an asylum.

**The Mechanism of Cerebral Injury by Contre-coup.**—Allen (*B. M. J.*, May, 1896) bases his theory of *contre-coup* on the fact that the brain having a higher specific gravity than the cerebro-spinal fluid, it will always rest on that surface of the cranium which at the moment happens to be downward, the cerebro-spinal fluid accumulating on the upward side. As injuries usually occur to the skull at the part which is downwards, that part, he says, receives the protecting support of the skull, while the opposite point, being unsupported, sustains damage.

### SURGERY OF THE SPINE.

**Lumbar Puncture.**—In spite of the very extensive trial which has been given on the Continent to this lumbar puncture of the vertebral theca as a diagnostic and therapeutic measure in cases of increased intra-cranial tension, observers seem to find difficulty in coming to an agreement as to its real value. It is true that a majority set a high value on the procedure as a diagnostic step. For example, Krönig (*Med. Week*, April, 1896) says that Lichtheim and Fürbringer found the tubercle bacillus in 80 to 90 per cent. of cases of tubercular meningitis, and that he himself has found it in 100 per cent. Cohn (*Med. Week*, May, 1896) has also got positive results in the same disease; and Gaibissi (*Gazz. degli Œped.*, Feb., 1896) produced tubercular meningitis in a rabbit by injecting the fluid withdrawn from a suspected case. The same observer has found staphyloccocci and Fraënkel's diplococcus in other cases. Stadelman (*Med. Week*, May, 1896) also found the pneumococus in a case of pleuro-pneumonia. On the other hand Lenhartz (*Münch Med. Woch.*, Feb., 1896) only found the tubercle bacillus in one out of twelve cases in which fluid was examined; and in one of three cases of cerebro-spinal meningitis he failed to find the streptococcus, although it was demonstrable in the fluid after death. Goldscheider (*Med. Week*, April, 1896) in a large number of cases has failed to find the tubercle bacillus, not only in the fluid withdrawn during life, but also after death. Stadelman (*Berlin Klin. Woch*, No. 27, 1895), too, was unable to discover organisms in a case of purulent meningitis following basal fracture, and in another resulting from septic middle ear disease, staining and culture methods alike proved negative.

Lenhartz places no diagnostic value on the specific gravity of the fluid withdrawn; but he considers a low percentage of albumen (*e.g.*, ·025 per cent.) indicative of an inflammatory condition, while higher proportions (2 to 4 per cent.) point to tumour or serous apoplexy.

In this he agrees with Quincke. In meningitis, as a rule, the fluid is clear, in traumatisms and in some cases of tubercular meningitis it is blood-stained.

There is even a less confident expression of opinion as to the therapeutic value of the measure forthcoming. Some writers in recording cases make no mention of the results beyond the aid it gives in diagnosis. Recourse has been had to lumbar tapping in a great variety of ailments, *e.g.*, in the tubercular, serous, and epidemic cerebro-spinal forms of meningitis, in chronic hydrocephalus, in acute œdema of the brain following injuries, in tumours, and in chlorosis.

Lenhartz was satisfied with the results in cases of acute serous meningitis, cerebral œdema of traumatic origin, and in chlorosis. Out of thirty cases of severe chlorosis, in twenty-nine subsidence of the headache and improvement of the general condition followed at once on the tapping. König and Krauze also have observed marked benefit to the symptoms in cases of meningitis, cerebral tumour, and chlorosis.

Giabissi can only say that "no evil results followed the operation," an opinion which Goldscheider does not go beyond. Schultze (*Med. Week*, May, 1896) considers the results too ephemeral to justify the risks of the procedure. That it is attended with a certain amount of risk is shown by the fact that cases of death following rapidly on its use have been reported by Schultze (six hours after), Krönig (three minutes after), Lenhartz (seven hours after), and others.

Another point against the operation is that recovery has followed in cases where unsuccessful attempts to withdraw fluid have been made.

**Lumbar Puncture of Intra-dural Hæmatoma.**—Kiliani (*N. York Med. Jour.*, March, 1896) gives an extended account of one of the only two cases on record of tapping for sub-dural hæmatoma. A man, forty-five, fell twenty feet, and was found partly conscious some hours after. He showed typical signs of injury to the cord in the lumbar region and cauda equina. The third lumbar spine was excessively tender. No improvement took place with extension and counter-extension, and in a week a puncture was made in the lumbar region and 8 c.cm. of thickish tar-coloured blood removed. Within an hour the anæsthesia began to pass off and gradually improved. Slight muscular power also returned. About a month later the patient died suddenly with symptoms of asphyxia. No post-mortem examination was granted.

**[illegible]ngocele and Spina Bifida**—The operative treatment and after results of two cases of meningocele are reported by Tilman (*Berlin Klin. Woch.*, 48, 1895). (1) Child, three weeks old. Meningocele size of walnut, translucent. An incision was made, the pedicle ligatured, and the sac cut away. The wound healed perfectly. Fifteen months after operation a hole the size of a pin's head was found under the cicatrix, and in the subcutaneous tissue a myxomatous mass with lymphatics and blood vessels, and a fine cord and delicate membrane ran through the space. (2) In a child, a year old, a large translucent tumour was found in the occipital region, with the skin over it very thin and vascular. The skull was hydrocephalic. There was no optic atrophy, but marked venous congestion. On operating a litre of fluid was drawn from the cavity, the pedicle was clamped and ligatured, and the incision closed. After fourteen days of health vomiting and diarrhœa began, with cramp in the extremities, nystagmus, right-sided facial paralysis, and paralysis of the diaphragm. Death took place twenty-three days after operation. On post-mortem examination there was no meningitis. In the squamous temporal bone was a hole admitting the tip of the little finger, through which the meningocele had projected. There was marked internal hydrocephalus. The wall of the sac was very tubercular in the inside, was adherent to the skin, and was lined with a thick fibrous membrane containing elastic tissue and a delicate vascular layer internally. Tilman says that in operating it is essential to exclude the possibility of brain matter being in the sac, and for this purpose the light test is useful. It is also important to avoid the loss of blood as far as possible.

Heath (*Practitioner*, July, 1896) in a clinical lecture describes some cases of meningocele and encephalocele seen by himself and others.

Two cases of spina bifida successfully cured by ligature of the pedicle in patients aged respectively thirty-two and six years, are reported by Thomas (*Lancet*, May, 1896), and other cases are recorded by Dalziel (*Glas. Med. Jour.*, July, 1896) and Nicoll (*Glas. Med. Jour.*, July, 1896).

**Injuries to the Spine.**—The value of early reduction of fracture-dislocations of the cervical vertebræ is illustrated by two independent cases published by American surgeons: (1) Nammack (*N. Y. Med. Rec.*, July, 1896) was called to a lad, aged twenty-one, who had struck his head against the bottom on diving into shallow water. Above the fifth cervical spine a marked hollow existed, the head being drawn back and fixed. From the mouth a distinct projection forwards could be felt. No paralysis of motion or sensation was present. Reduction was easily effected by extension and counter-extension under ether anæsthesia, and the parts were retained in position by a plaster of Paris case. In five weeks recovery was complete. (2) The other case was treated by Paine (*N. Y. Med. Rec.*, May, 1896). A man of fifty fell from a waggon, and the same physical signs as in the last case were present, and in addition a fragment of broken bone (the right transverse process of the fourth cervical vertebra) could be detected loose. Extension and counter-extension under chloroform failed to effect reduction, but by firm pressure through the mouth the bones were readily replaced. At the moment of reduction the breathing stopped and the pupils dilated, but ordinary measures restored the patient, who recovered, and four and a half years later showed no abnormal sign save a slight rigidity of the neck.

Several cases of injury treated by laminectomy have been reported by Chipault (*Rev. de Chir.*, 1896). One was operated on eleven days after an injury causing compression of the cord by a fragment of the body of the eleventh dorsal vertebra. After removal of the fragments recovery gradually took place, and two and a half years later patient was quite free of any trouble, save some weakness of the bladder and anus. In another case of subluxation forward and to the right of the fourth and fifth cervical vertebræ, paralytic and sensory disturbances resulted. Thirteen months after the accident the spine was exposed, and the fourth vertebra fixed into position with silver wire. Two years later all was well. Wyeth (*N. Y. Med. Rec.*, May, 1896) reports the results of operative treatment in a series of traumatic cases. The result was only entirely satisfactory in one out of seven cases operated upon. Parona (*Ep. B. M. J.*, May, 1896) exposed the eighth dorsal spine for neuralgia following an injury, and, finding no dis-displaced bone, stretched the nerves close to their foramina of exit, with the result that the pain very rapidly and completely disappeared.

It is obvious that the main element on which success depends in these cases is the condition of the cord, a point which it is extremely difficult to determine before operation. It is interesting to observe from Enderlen's (*Deutsch Zeitschrift-f-Chir.*, *Bd.* xl.) experiments that the injection of fresh blood, and also the introduction of small particles of kidney substance under the dura determines degeneration of the cord. He attributes this not to the pressure exerted, but to interference with circulation. Similar results may follow in traumatic cases, hence the importance of giving a guarded prognosis before operating.

**Neuralgia of the Spine.**—Illustrations of the effect of different methods of treating this very troublesome affection are offered in the cases of Parona (*Ep. B. M. J.*, May, 1896) and Chipault (*Ep. B. M. J.*, May, 1896). In the case of the former the disease involved the seventh and eighth dorsal nerves on the left side, and was supposed to be of rheumatic origin. Fifteen-grain doses of potassium iodide injected deeply into the tissues in the region of the affected nerves gave relief when every ordinary method had failed. Eight injections sufficed. In the other case the posterior nerve roots corresponding to the last two cervical and first dorsal vertebræ were resected with good results, for persistent neuralgia of certain parts of the right upper extremity. A third case has above been referred to under injuries to the spine.

Bremer (*Med. Rec.*, August, 1896), vigorously attacks those who resort to operative measures in the treatment of coccygodynia, and cites illustrative cases.

**The Treatment of Spinal Caries.**—Attempts have from time to time been made to get over the necessity of confining the sufferers from tubercular caries of the spine to bed during the long drawn-out treatment of this disease. Lovett (*Med. News*, 1,207, 1896), from a study of the subject, comes to the conclusion that all forms of apparatus devised to permit of what he calls "ambulatory treatment," involve a much greater risk of failure than the orthodox treatment by rest in bed with extension, and the onus of such should be left to the patient or his friends, if they insist upon his going about.

The results of laminectomy in spinal caries is not very encouraging. Neve (*Lancet*, April, 1896), contributes two new cases, one successful the other not; Cotterell (*Lancet*, March, 1896) one, only partially successful; and Paton (*Lancet*, May, 1896) one in which temporary improvement took place, but the child died later, and on post-mortem examination it was found that the cord was almost completely destroyed by pressure. Wyeth had a complete recovery after removal of a tubercular neoplasm by laminectomy; and Parona one in which local recovery took place, but the patient died of pulmonary phthisis soon after.

**Diagnosis of Spinal Caries.**—Noble Smith (*B. M. J.*, June, 1896) reports, with more or less convincing illustrations, two cases, in one of which the diagnosis of tubercular caries was negatived, and in the other confirmed by the use of Roentgen rays.

**Tumours of the Spinal Cord.**—Kummell (*Ann. Surg.*, July, 1896) adds a new case of operation for spinal tumour. A man, aged forty-seven, suffered from a soft sarcomatous tumour of the sacrum, which was removed by operation, and in three months the patient resumed his work. About a year later however, evidence of a secondary tumour, in the form of paralysis and anæsthesia referable to the dorsal region of the spine, appeared and gradually increased. After a few months a second operation was performed, and a tumour about the size of a small apple was removed. It had been compressing the left side of the cord, which it had reduced to less than half its size. Immediately the tumour was removed the cord regained its natural size and colour. In sixteen days motion began to return, and in three months he was able to return to work, suffering still, however, from incontinence of urine and some rectal difficulty.

